# Effects of human serum and apo‐Transferrin on *Staphylococcus epidermidis* RP62A biofilm formation

**DOI:** 10.1002/mbo3.379

**Published:** 2016-05-16

**Authors:** Pengfei She, Lihua Chen, Yong Qi, Huan Xu, Yuan Liu, Yangxia Wang, Zhen Luo, Yong Wu

**Affiliations:** ^1^ Department of Medicine Clinical LaboratoryThe Third Xiangya Hospital of Central South UniversityChangsha410013China; ^2^ Xiangya School of MedicineCentral South UniversityChangsha410013China

**Keywords:** Apo‐Transferrin, biofilm, crystal violet, serum, *Staphylococcus epidermidis*, XTT.

## Abstract

Biofilm‐associated *Staphylococcus epidermidis* infections present clinically important features due to their high levels of resistance to traditional antibiotics. As a part of human innate immune system, serum shows different degrees of protection against systemic *S. epidermidis* infection. We investigated the ability of human serum as well as serum component to inhibit the formation of, and eradication of mature *S. epidermidis* biofilms. In addition, the synergistic effect of vancomycin combined with apo‐Transferrin was checked. Human serum exhibited significant antibiofilm activities against *S. epidermidis* at the concentration without affecting planktonic cell growth. However, there was no effect of human serum on established biofilms. By component separation, we observed that antibiofilm effect of serum components mainly due to the proteins could be damaged by heat inactivation (e.g., complement) or heat‐stable proteins ≥100 kDa. In addition, serum apo‐Transferrin showed modest antibiofilm effect, but without influence on *S. epidermidis* initial adhesion. And there was a synergistic antibiofilm interaction between vancomycin and apo‐Transferrin against *S. epidermidis*. Our results indicate that serum or its components (heat‐inactivated components or heat‐stable proteins ≥100 kDa) could inhibits *S. epidermidis* biofilm formation. Besides, apo‐Transferrin could partially reduce the biofilm formation at the concentration that does not inhibit planktonic cell growth.

## Introduction

Bacterial biofilms are organized communities containing lots of densely packed cells (Prindle et al. 2015). Microorganisms within biofilms are less susceptible to antimicrobial treatment than their planktonic counterparts (Nuryastuti et al. 2009). The hardiness of biofilms and their resistance to current antibiotics as well as host immune clearance mechanisms has led to a growing problem in healthcare settings (Nithyanand et al. 2015). *Staphylococcus epidermidis*, a member of the group of coagulase‐negative staphylococci, belongs to the commensal skin flora of every human individual (Chen et al. 2015). *Staphylococcus epidermidis* is today one of the most important bacteria related to hospital acquired infection, and has been known for many years as a leading cause of infections related to indwelling medical devices such as vascular catheters, prosthetic joints, and artificial heart valves (Vuong and Otto 2002; Hall‐Stoodley et al. 2004). This is related, in part, to the organism's ability to adhere to biotic or abiotic surfaces and form biofilms (Oliveira et al. 2007).

Although some plasma proteins such as fibronectin, fibrinogen, and thrombospondin have a propensity to enhance bacterial adhesion to surfaces coated with these proteins (Hermann et al. 1991; Nilsson et al. 1998; Chia et al. 2000), recent studies have revealed a strong inhibition of biofilm formation on biomaterials in the presence of whole serum (Ardehali et al. 2002; Ardehali et al. 2003; Abraham and Jefferson 2010; Hammond et al. 2010; Naves et al. 2010; del Prado et al. 2010; Samaranayake et al. 2013; Ding et al. 2014; Wuren et al. 2014). These observations prompted an investigation of the role of serum proteins, for example, transferring (Ardehali et al. 2003; Artini et al. 2012), in biofilm inhibition.

However, there is scant systematic research about the role played by human serum (HS) or its constituents in *S. epidermidis* biofilm growth. Although some studies found that apo‐Transferrin (apo‐Tf, the iron depleted form of Transferrin) but not holo‐Transferrin (apo‐Tf, the iron loaded form of Transferrin) do have ability to inhibit bacterial adhesion (Ardehali et al. 2003; Artini et al. 2012), there is no evidence whether there is a synergistic effects when combining the apo‐Tf with vancomycin.

To determine the effect of human serum on the development of *S. epidermidis* biofilms, we characterized planktonic as well as biofilm growth with and without human serum. Then, we determined which serum components play a leading role in biofilm inhibition by removing or dividing the serum protein component. In addition, we verified the effect of transferrin on *S. epidermidis* biofilm inhibition, and evaluated the synergistic effects of transferrin combined with vancomycin.

## Materials and Methods

### Bacterial strains, growth media, and reagents

*Staphylococcus epidermidis* RP62A (ATCC35984) was kindly provided by Di Qu (Laboratory of Medical Molecular Virology, Shanghai Medical College, Fudan University, Shanghai, China). Pure stock cultures were maintained at −80°C in 30% (vol/vol) frozen glycerol solution. Bacteria were subcultured on blood agar plates overnight at 37°C. LB medium was used for bacterial culture in all experiments unless otherwise specified. Human albumin, apo‐Tf, and holo‐Tf (Sigma‐Aldrich, St. Louis, MO) were used and dissolved in distilled water. The absorbance (A) was measured by a spectrophotometer (Bio‐Tek, USA).

### Preparation of human serum

Human blood was donated by volunteers, who reported that they took no medication of any type for at least 10 days before the venipuncture. Serum was separated from the solid clot and was then centrifuged at 1200*g* for 15 min, and the supernatant was passed through a sterile syringe filter (Millipore Millex‐GP, USA) with pore size of 0.22 *μ*m. And the filtrate was stored at −20°C until further use.

### Biofilm determination

To detect the inhibitory effect of HS or Transferrin (apo‐Tf and holo‐Tf) on biofilm formation of *S. epidermidis* RP62A, the semiquantitative determination of biofilm formation was performed in microtiter plates as described earlier, with minor modifications (Nesse et al. 2015). Four *μ*L culture and 196 *μ*L LB with or without the HS or Transferrin to be tested were added to each well in 96‐well cell culture plates (Corning/Costar, USA), and incubated at 37°C for 24 h. And 1.5 mg/mL bicarbonate was added to Transferrin to eliminate the possible iron of LB prior to usage when needed. Biofilm formation was determined by a crystal violet (CV) and XTT assays. Crystal violet was used as indicator of total biofilm biomass. Briefly, biofilm was washed gently and stained with 0.5% (w/v) crystal violet solution. After staining, the plates were washed again and 200 *μ*L of 95% ethanol was added to dissolve stained dye, the absorbance was measured at 570 nm. XTT can be converted to a colored formazan salt in the presence of metabolic activity, which was applied to determine antimicrobial susceptibility. Briefly, after exposure to HS or Transferrin, biofilms were washed, then 200 *μ*L of a solution containing 200 mg/L of XTT and 20 mg/L of PMS (phenazine methosulfate) was added to each well. The microtiter plates were incubated for 3 h at 37°C in the dark. The absorbance was measured at 490 nm (Gomes et al. 2009).

For the mature biofilm disassembly valuation, primarily the mature biofilm was allowed to form as follows: a volume of 100 *μ*L of 1:200 diluted overnight bacterial culture was added to a 96‐well cell culture plate with LB and the plate was incubated at 37°C for 24 h. Later the wells were washed with 0.9% saline and HS was incorporated into LB to different concentrations. The plate was incubated for another 24 h and biofilm biomass was determined by CV and XTT assays as described earlier.

### Planktonic cell growth

The planktonic cell growth assay was modified from Huang and Li as described previously (Huang et al. 2014). Overnight grown *S. epidermidis* RP62A (optical density at 630 nm was adjusted to 0.45 with LB; 5 × 10^7^ CFU/mL) cells were diluted 1:100 in LB with 0%, 5%, 10%, 25%, and 50% HS, and at the same time we set 3 *μ*g/mL vancomycin (the MBC of *S. epidermidis* RP62A) as a positive control. These samples were then grown in 50 mL centrifugal tubes (Corning/Costar, USA) at 37°C with agitation (160 rpm) for 6, 12, and 24 h, respectively. After incubation, 200 *μ*L culture medium was carefully transferred to another microtiter plate. The planktonic culture turbidity was read at 630 nm by a spectrophotometer.

### HS component separation

Heat‐inactivated serum was prepared by heating at 56°C for 30 min to inactivate complement and other heat‐inactivated components (HS‐Heated) (Ayache et al. 2006). Proteinase K‐treated serum (HS‐Proteinase) was prepared by incubating with 25 mg/mL proteinase K at 58°C for 1 h followed by incubation at 85°C for 1 h to inactivate the protease. On the other hand, proteins in HS were separated into two fractions, one containing proteins ≥100 kDa and the other containing proteins <100 kDa, using Amicon^®^ Ultra‐4 centrifugal filter devices (Millipore). All fractions were filter sterilized (0.22 *μ*m pore size filter) (Ding et al. 2014). Positive and negative controls were prepared in LB with (5%) or without HS, respectively. The effects of these serum components on *S. epidermidis* formation were valuated by CV and XTT assays as described earlier.

### Effect of HS and apo‐Tf on *S. epidermidis* biofilm morphology

The cover slides were disinfected by soaking them into 75% ethanol for 30 min, followed by successive washes with distilled water using a syringe. After drying, the cover slides were placed into 6‐well cell culture plates with 2 mL HS or apo‐Tf at designed concentration containing 40 *μ*L overnight grown *S. epidermidis* (optical density at 630 nm was adjusted to 0.45 with LB). After 24 h incubation, the culture media was removed and the cover slides were washed three times with 0.9% saline, then the cover slides were stained with CV as described earlier. The biofilm was visualized and photographed with a light microscope (Olympus CX31, Tokyo, Japan) at 10 × 40 magnification. For mature biofilm disassembly valuation, 24‐h biofilms were treated with or without HS for another 24 h, and were photographed with the microscope.

### Initial adhesion assay

Experiment was conducted based on the method previously described by Santiago et al. (2015). A 96‐well microtiter plate was prepared with apo‐Tf at the concentration of 5 mg/mL. Aliquots of *S. epidermidis* suspension (adjusted to 0.5 McFarland) were added to these wells. The plate was incubated for 2 h at 37°C. Following incubation, the wells were washed with saline, and biomass of cell attachment was determined by CV assay as described earlier.

### Synergy testing of apo‐Tf with vancomycin

*Staphylococcus epidermidis* strain (1 × 10^5^ CFU/200 *μ*L) in LB was incubated at 37°C for 24 h with sub‐MIC VAN (0.0313 *μ*g/mL) and apo‐Tf (4 mg/mL) alone and in combination in 96‐well cell culture plates and the biomass of biofilm cells was measured by CV assay as described earlier.

### Statistical analysis

Data were described as mean ± SD. Differences between groups were examined for statistical significance using Student's *t* test or one‐way analysis of variance. All statistical analyses were performed by Graph Pad Prim 6.0 software. The data means were considered to be different at *P *<* *0.05. All experiments were performed in triplicate and repeated three times.

## Results

### HS treatment reduces biofilm formation, but has no effect on established biofilms

**Figure 1:**
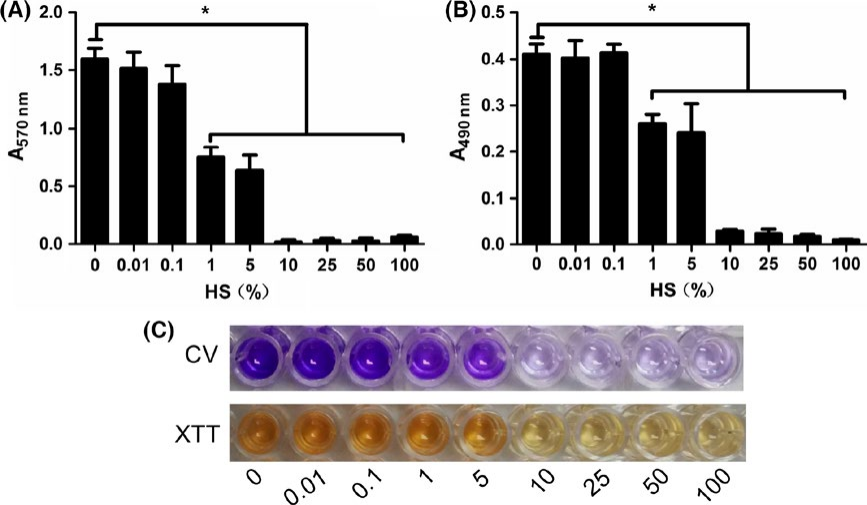
Human serum (HS) inhibits *Staphylococcus epidermidis* ATCC35984 biofilm formation. Overnight culture was treated with 0–100% HS at 37°C for 24 h. The volume of biofilm was determined by CV assay (A, C) and by XTT assay (B, C). *P <* *0.05 versus untreated control, and error bars indicate SD. CV, crystal violet.

**Figure 2:**
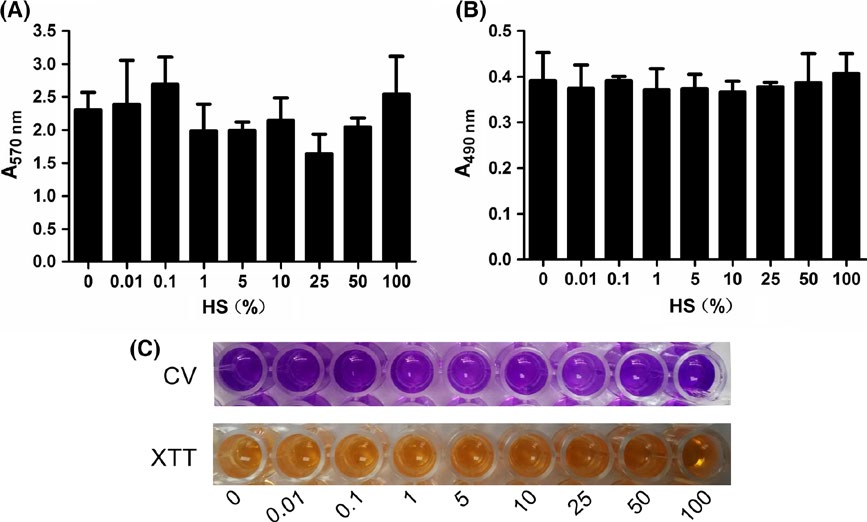
Human serum (HS) has no effect on *Staphylococcus epidermidis* ATCC35984 mature biofilm eradication. Twenty‐four‐hour mature biofilms were cultured overnight with HS at concentrations ranging from 0% to 100% at 37°C for another 24 h and the quantification of biofilm biomass determined by CV assay (A, C) and by XTT assay (B, C). Error bars indicate SD. CV, crystal violet.

**Figure 3:**
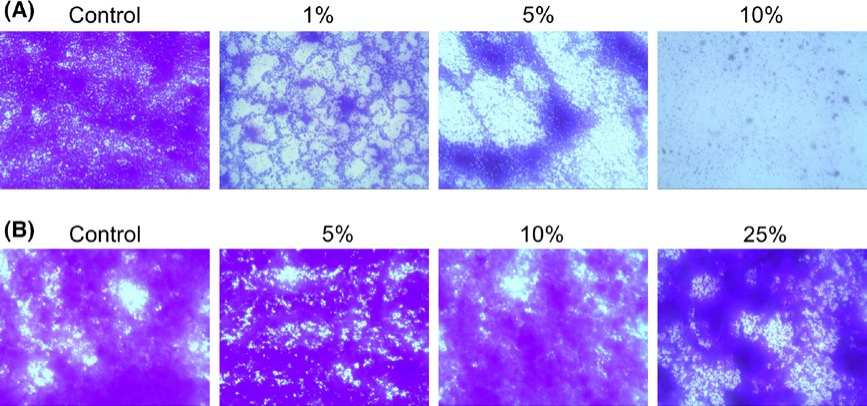
Optical micrographs showing the effects of human serum (HS) on *Staphylococcus epidermidis* ATCC35984 biofilm formation (A) and eradication (B). Representative images (magnification, 10 × 40) of biofilm was treated with different concentrations of HS, and the effects of HS were assessed by crystal violet staining.

The previously established biofilm assays (CV and XTT assay) were used to evaluate the ability of HS to prevent biofilm formation and disperse established *S. epidermidis* biofilms. CV assay showed that HS started to inhibit *S. epidermidis* biofilm formation at the concentration ≥1%. More specifically, in the presence of 1–5% HS, biofilm formation was reduced to 50–60% compared to control. And HS almost completely inhibit *S. epidermidis* biofilm formation at the concentration ≥10% (Figs. 1A and C and 3A). In accordance with the CV assay, quantitative analysis using XTT assay revealed that HS at the concentration ≥1% significantly attenuated metabolic activity due to biofilm inhibition by HS (Figs. 1B and C and 3A). We then tested the effects of HS on 24‐h mature biofilm, but it had no effect on established biofilms in our assay conditions up to 100% (Figs. 2A–C and 3B).

### Effect of HS on planktonic growth of *S. epidermidis*

**Figure 4:**
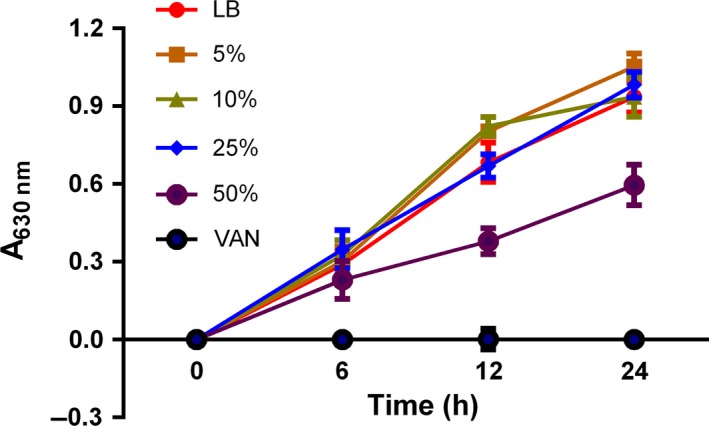
Planktonic cell growth of *Staphylococcus epidermidis* ATCC35984 in the presence of human serum (HS). As a control, the concentration of VAN is 3 *μ*g/mL which is equal to the MBC we tested in this study. Results are representative of three experiments, error bars indicate SD.

Figure 4 depicts the effect of different HS concentrations on planktonic cells of *S. epidermidis* RP62A. HS concentrations below 25% did not affect the planktonic cell growth, concentrations greater than 50% partially inhibited bacterial growth, which confirmed that inhibition of biofilm formation was not due solely to growth inhibition.

### Characterization of the inhibitory components

**Figure 5:**
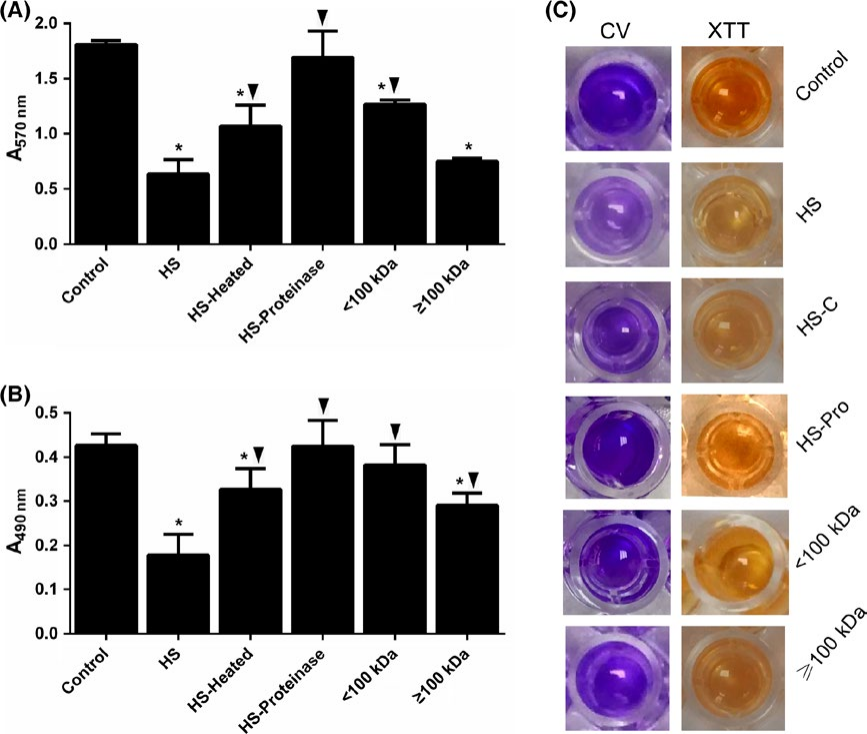
The inhibitory effect of human serum (HS) on *Staphylococcus epidermidis* ATCC35984 biofilm can be fractionated. Complement (C) and protein (Pro) were removed from HS, respectively. Besides, HS was divided into two fractions, one containing proteins ≤100 Da and the other containing proteins >100 kDa. The effects of those fractions on the formation of *S. epidermidis* biofilm were examined by CV assay (A, C) and by XTT assay (B, C). *P <* *0.05 versus untreated control; ▼*P *<* *0.05 versus 5% HS‐treated group, and error bars indicate SD. CV, crystal violet.

To further study the components of HS that inhibit the biofilm formation of *S. epidermidis*, we heated the serum at 56°C for 30 min to inactivate heat‐unstable components (Ayache et al. 2006). We found that the removal of heat‐unstable components in serum reduced ~ 43.48% (for CV assay) and ~57.14% (for XTT assay) inhibitory activity of serum, respectively, when compared with 5% HS‐treated group, which suggests that heat‐unstable components (e.g., complement) contributes modestly to the inhibition of *S. epidermidis* biofilm formation. Then, to investigate whether the rest of the part of the inhibitory activity is due to other heat‐stable proteins in serum, proteinase K was used to degrade proteins in the heat‐inactivated HS, and the inhibitory activity completely disappeared. We then separated the HS proteins by a membrane fractionation. In this process, the traction retained by the membrane contains all proteins with a MW of ≥100 kDa, while the pass‐through fraction contains all proteins with a MW of <100 kDa. As shown in Figure 5, most inhibitory effect of HS resides within the fractions containing proteins ≥100 kDa. However, the fraction containing proteins of <100 kDa still produced a significant reduction. Together, these results support the possibility that HS contains multiple factors that interfere with *S. epidermidis* biofilm formation.

### Effect of transferrin on *S. epidermidis* biofilm formation

**Figure 6:**
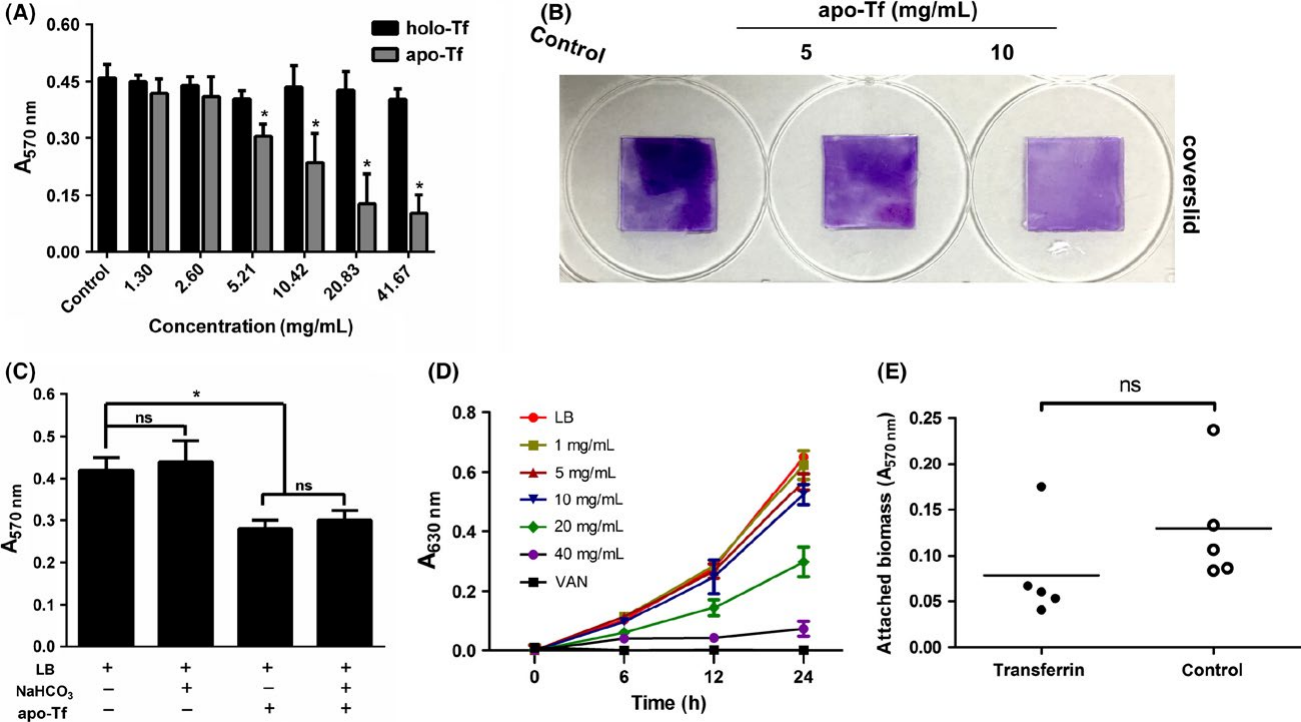
Effects of the HS component transferrin on *Staphylococcus epidermidis* ATCC35984. (A) Biofilm formation by *S. epidermidis* in the presence of apo‐Tf and holo‐Tf at the concentration ranging from 0 to 41.67 mg/mL. *P <* *0.05 versus untreated control, and error bars indicate SD. (B) Apo‐Tf can inhibit biofilm formation on coverslips. (C) Effects of bicarbonate on the inhibitory effect of apo‐Tf. (D) Effects of apo‐Tf on *S. epidermidis* planktonic cell growth. Error bars indicate SD. (E) apo‐Tf has no effects on bacterial attachment. ns, *P > *0.05. HS, human serum

Once it was determined that apo‐Tf was the major component in the protein pool of serum exhibiting the inhibitory activity (Ardehali et al. 2003), biofilm inhibition assay (CV assay) was performed in the presence of purified protein apo‐Tf and holo‐Tf. The inhibitory effect of apo‐Tf was diminished at the concentration <5.21 mg/mL. The presence of apo‐Tf at the concentrations between 5.21 and 41.67 mg/mL yielded dose‐dependent inhibition of *S. epidermidis* biofilm in this concentration range, however, holo‐Tf cannot inhibit *S. epidermidis* biofilm formation even at high dose (Fig. 6A and B). On the other hand, adding bicarbonate (1.5 mg/mL) into apo‐Tf cannot change the inhibitory effect of apo‐Tf indicating that the iron ion in the LB may have no impact on the inhibitory effect of apo‐Tf (Fig. 6C). In consistence of the results above, the inhibition of biofilm formation was not due solely to growth inhibition. As is shown in Figure 6D, time–growth curves indicated that the presence of apo‐Tf at the concentration ≤10 mg/mL did not significantly affect the growth of *S. epidermidis*. So we chose 5 mg/mL apo‐Tf as a measure of the inhibitory effect of apo‐Tf on *S. epidermidis* bacterial initial adhesion. Although apo‐Tf showed significant inhibitory effect on biofilm formation of *S. epidermidis*, it had no inhibitory effect on bacterial initial adhesion (Fig. 6E). These results together would suggest that the inhibitory effect of HS on *S. epidermidis* biofilm formation was not mainly, but partially due to the presence of apo‐Tf; however, it had no effect on initial bacterial adhesion.

### Synergistic effect between VAN and apo‐Tf on *S. epidermidis* biofilm formation

**Figure 7:**
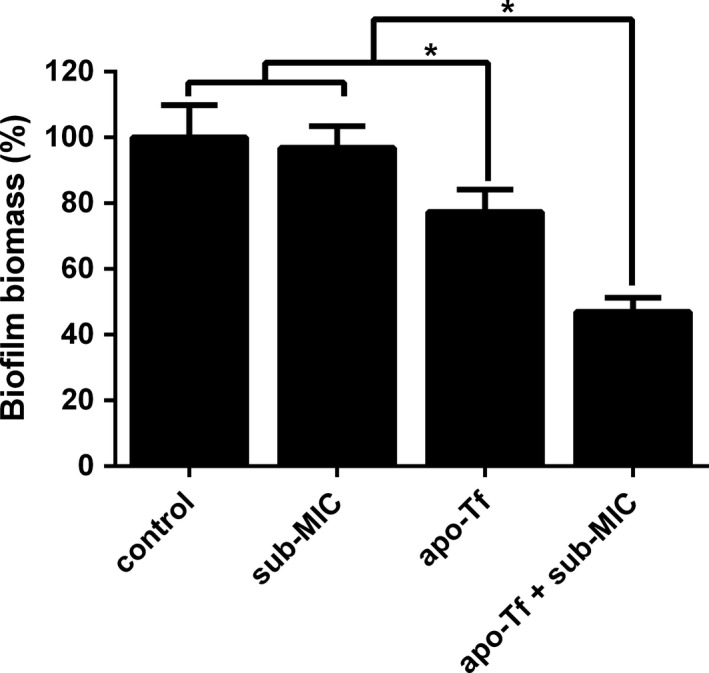
Effect of VAN with and without transferrin on biofilm formation of *Staphylococcus epidermidis* ATCC35984. The graph presents biofilm biomass (%) for the strain grown in LB broth (control) or in LB broth amended with subinhibitory concentrations (sub‐MIC) of VAN or apo‐Tf (4 mg/mL) or both of them (apo‐Tf + sub‐MIC). Statistically significant differences are indicated for each sample treated with apo‐Tf alone or apo‐Tf combined with sub‐MIC VAN compared to the control and sample treated with sub‐MIC VAN only (*P <* *0.05). Error bars represent SD.

To assess the synergistic effects between VAN and apo‐Tf on *S. epidermidis* biofilm formation, *S. epidermidis* was exposed to combinations of these agents and their viability was examined by CV assay. The result of the combination assay was presented in Figure 7. We found that VAN at subminimal inhibitory concentration (0.0313 *μ*g/mL) almost had no effect on *S. epidermidis* biofilm formation (*P *>* *0.05). However, apo‐Tf could cause ~20% reduction in biofilm formation at the concentration of 4 mg/mL. We then tested whether there was a synergistic effect between VAN and apo‐Tf on *S. epidermidis* biofilm formation. Following 24 h of apo‐Tf combining sub‐MIC VAN treatment, there was ~43% reduction in biofilm formation compared to the only sub‐MIC VAN treatment group. These results suggest that there was a synergistic effect on inhibition of *S. epidermidis* biofilm formation between sub‐MIC VAN and apo‐Tf.

## Discussion

To make the transition from a commensal organism to a systemic pathogen, *S. epidermidis* must first enter the blood stream. In such circumstance, *S. epidermidis* is exposed to the innate immune defenses. As a part of human innate immune system, serum and its components show different degrees of protection against systemic *S. epidermidis* infection (Kojic and Darouiche 2004; Ding et al. 2014).

In this work, we demonstrated that HS blocks *S. epidermidis* RP62A biofilm formation at concentrations without limiting bacterial growth. And fractionation analysis indicates that the major components that interfere with biofilm formation are heat‐inactivated components and heat‐stable proteins ≥100 kDa in size. We then tested the antibiofilm effect of apo‐Tf, which was reported to be the most important component in biofilm inhibition (Ardehali et al. 2003). Although the effects of apo‐Tf on *S. epidermidis* RP62A biofilm formation was similar to that reported by other investigations (Ardehali et al. 2003; Artini et al. 2012), higher concentrations of apo‐Tf were required in our experiment; besides, apo‐Tf shows no effect on *S. epidermidis* initial attachment at the antibiofilm concentration. We further showed that apo‐Tf can potentiate VAN susceptibility in biofilm inhibition. To our knowledge, there are no studies demonstrating the synergistic antibiofilm effect between apo‐Tf and VAN, which could be used to guide the use of antibiotics.

Previous studies found that some plasma proteins have the ability to promote biofilm formation (Hermann et al. 1991; Nilsson et al. 1998; Chia et al. 2000), but recent studies have investigated a strong inhibition of adherence of various bacterium to biotic or abiotic substances in the presence of whole serum (Ardehali et al. 2002, 2003; Abraham and Jefferson 2010; Hammond et al. 2010; Naves et al. 2010; del Prado et al. 2010; Samaranayake et al. 2013; Ding et al. 2014; Wuren et al. 2014). However, the effects of serum on yeast strains are varied. In pioneering investigations, serum could promote the growth of various *Aspergillus* spp. (Wuren et al. 2014); besides, abiotic surfaces preconditioned with body fluids such as serum and saliva promote increased yeast colonization and biofilm growth of *Candida albicans* (Nikawa et al. 2000; Samaranayake et al. 2013) and potentiates the expression of genes associated with antifungal drug resistance (Samaranayake et al. 2015), but some studies found that serum could inhibit *C. albicans* biofilm formation (Ding et al. 2014). In consensus with previous studies in bacteria, we demonstrate that HS could inhibit biofilm formation in *S. epidermidis*.

**Figure 8:**
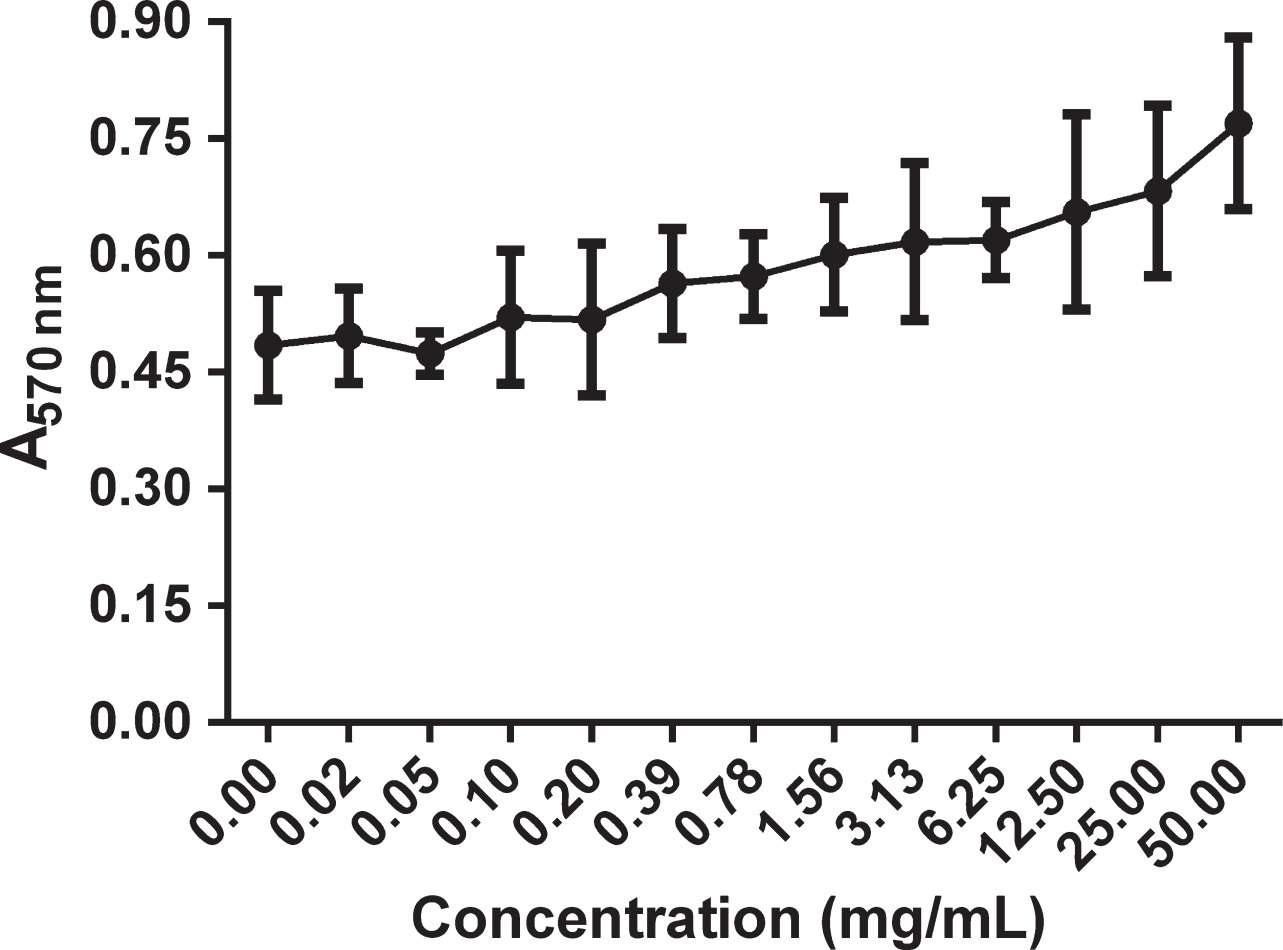
Effect of albumin on *Staphylococcus epidermidis* ATCC35984 biofilm formation. Error bars indicate SD.

In search of the inhibitory component, serum was fractionated and analyses of the protein pools indicated that the major portion of the biofilm inhibitory activity of serum appears to be heat‐inactivated components and proteins ≥100 kDa in size. The heat‐inactivated components contains complement and some soluble factors (Ayache et al. 2006), and the ≥100 kDa fraction mainly contains immunoglobulins (150 kDa), C‐reactive protein (105 kDa), and ceruloplasmin (150 kDa) (Hammond et al. 2010). Moreover, Abraham and Jefferson (2010) found that a low‐molecular‐weight component of HS inhibits biofilm formation in *Staphylococcus aureus*, and the component was protease resistant and heat stable. A similar circumstance was previously described by Hammond et al. (2010), who examined the effects of HS on biofilm formation by *Pseudomonas aeruginosa*, and they described that serum albumin played a critical role in inhibition of PAO1 biofilm formation. Although our studies found that both complement and total protein of serum had inhibitory effect on *S. epidermidis* biofilm formation, there was no effect of serum albumin on *S. epidermidis* biofilm formation (Fig. 8) probably because of the different strains and culture conditions and serotypes sources. To confirm this hypothesis, future studies are needed to identify this component of human serum.

Within the body, most of the extracellular iron is complexed by the serum glycoprotein, transferrin, that serves to transport iron from areas of supply to areas of need; transferrin is one of the most common serum component, is a monomeric glycoprotein structurally divided in two lobes, each capable of binding one iron III ion. The iron depleted form is named apo‐Tf, while the iron loaded form is named holo‐Tf (Adlerova et al. 2008). Transferrin has been shown to exert both a bacteriostatic and bactericidal effect in vitro on a variety of pathogens (Adlerova et al. 2008; Artini et al. 2012), and some investigations have revealed that transferrin has the effects on bacterial adhesion inhibition (Ardehali et al. 2003; Artini et al. 2012), but the inhibition of bacterial adhesion by serum is largely due to the presence of apo‐Tf, the iron deficient form of transferrin. On the contrary, the presence of holo‐Tf, the iron saturated form of transferrin, did not prevent bacterial adhesion to biomaterial surfaces (Brindle et al. 2011). Similar to the previous study, we found that apo‐Tf but not holo‐Tf could to some extent inhibit biofilm formation, but the higher concentration was required in vitro. On the other hand, we found that there are no effects of apo‐Tf on initial adherence of *S. epidermidis*. The reason for this could be the differences in the *S. epidermidis* strains used, and the incubation medium they used. Moreover, there are so many different components in the serum, and there may be a synergistic effect of apo‐Tf with other serum components in vivo, we cannot exclude the effect of apo‐Tf on inhibition of biofilm at physiological concentration.

There are two major steps in biofilm formation: (1) initial adhesion to biotic and abiotic substances and (2) cell–cell interaction (Mack et al. 2004). Initial adhesion and growth of microorganisms on the surface of an implant can be the most critical event in the development of device‐associated infection (Ardehali et al. 2003). The percentage of bacterial cell‐surface attachment is directly proportional to the final mass of biofilm formed. Although apo‐Tf was found to suppress *S. epidermidis* biofilm production, it has no effect on *S. epidermidis* initial adhesion.

Here, we demonstrate for the first time that there is a synergistic inhibitory effect on *S. epidermidis* RP62A biofilm formation for the combination of apo‐Tf with VAN. Antibiofilm activity in vitro depends on many factors such as the different strains used, culture conditions, and the medium used. Thus, we cannot draw any definitive conclusions on the basis of the results of this study. However, the new findings shown here should provide important clues for further study of the efficacy of combination therapy for biofilm‐forming *S. epidermidis*.

Taken together, our results indicate that in vitro HS significantly inhibit *S. epidermidis* biofilm formation due to the heat‐inactivated components and heat‐stable proteins <100 kDa in serum. Besides, apo‐Tf has partially antibioiflm effect and has synergistic effect with VAN in *S. epidermidis* biofilm inhibition. However, the inhibition profile of serum and its components under laboratory growth conditions may not be representative of in vivo conditions due to the complex and dynamic environment within the host. The observations of this study, however, will be useful as important base data for such future studies.

## Conflict of Interest

None declared.
